# Hydrogel Nanofilaments via Core-Shell Electrospinning

**DOI:** 10.1371/journal.pone.0129816

**Published:** 2015-06-19

**Authors:** Paweł Nakielski, Sylwia Pawłowska, Filippo Pierini, Wioletta Liwińska, Patryk Hejduk, Krzysztof Zembrzycki, Ewelina Zabost, Tomasz A. Kowalewski

**Affiliations:** 1 Department of Mechanics and Physics of Fluids, Institute of Fundamental Technological Research, Polish Academy of Sciences, Warsaw, Poland; 2 Faculty of Chemistry, University of Warsaw, Warsaw, Poland; University of California, San Diego, UNITED STATES

## Abstract

Recent biomedical hydrogels applications require the development of nanostructures with controlled diameter and adjustable mechanical properties. Here we present a technique for the production of flexible nanofilaments to be used as drug carriers or in microfluidics, with deformability and elasticity resembling those of long DNA chains. The fabrication method is based on the core-shell electrospinning technique with core solution polymerisation post electrospinning. Produced from the nanofibers highly deformable hydrogel nanofilaments are characterised by their Brownian motion and bending dynamics. The evaluated mechanical properties are compared with AFM nanoindentation tests.

## Introduction

In recent years polymer hydrogels have begun to be frequently used as biomaterials. Their structure remains insoluble in water due to crosslinking, while they are able to absorb large quantities of water (up to thousands of times their dry volume). Their swelling properties and softness resemble those of the extra-cellular matrix (ECM), thus making them an extremely attractive material for a large variety of biomedical applications, such as tissue culture, environment-sensitive drug delivery systems, and in various types of pharmaceutical products [1, 2].

Here we have demonstrated that the possibility to produce hydrogel filaments opens up new areas of applications where the fibrous structure is of crucial importance (e.g. nervous system, blood vessels, DNA encapsulation). Recently, nanostructured PNIPAAm/dsDNA composite hydrogels obtained in capillaries were successfully used as thermo-switchable systems with a potential application as biosensors for the detection of intercalators [3]. In the following text we show possibility to encapsulate BSA molecules into nanofilament structure, to construct either a highly flexible biosensor of intracellular environment or a dedicated drug carrier, more suitable then previously reported stiff nanofibres [4, 5].

It is well known that the mechanical properties of hydrogels play a crucial role in cell culture applications. These can be precisely tweaked by modifying their crosslinking density and thus their composition. Mechanical properties are even more crucial in the case of nanofilaments, where the high flexibility of nanoobjects is necessary for relocating them within a crowded intracellular environment. Predicting the behaviour of the deformable objects conveyed by the fluid flow is essential for understanding the physics of macromolecule suspensions, biofluids, and the industrial transportation of fibrous materials.

The flexible nanofilaments described here are characterised by deformability and elasticity resembling long DNA chains. We introduce a unique method for their fabrication based on the well known core-shell electrospinning technique [6, 7]. To the authors’ knowledge, this is the first method to use post electrospinning free radical polymerisation to obtain hydrogel core in polymer shell. The described in literature core-shell methods include post polymerisation of oligomers by UV radiation [8] or core curing after electrospinning [9]. Other examples including PNIPAAm hydrogel presents production of polymer core and hydrogel shell [10, 11]. Eventually, PNIPAAm hydrogel is grafted in the form of brushes through the atom transfer radical polymerisation (ATRP) onto nanofibers surface [12–14]. We find the proposed electrospinning technique of producing gel-like filaments to be easily available and much simpler then the reported microfabrication [15], template-guided synthesis [16, 17], or radiation synthesis [18].

Characterisation of the mechanical properties of hydrogels is not an easy task even for bulk materials. This is mainly due to their extraordinarily low stiffness and high nonuniformity. Due to the hydrogel matrix interactions with the environment, and depending on the degree of cross-linking, the mechanical properties of hydrogel samples may vary greatly. In the case of nanofilaments with characteristic dimensions well below 100 nm, the classical methods of evaluating material stiffness with standard tools (AFM) are quite tedious and, for very soft materials usually rather inaccurate. Therefore, for the assessment of the mechanical properties of our hydrogel filaments we used additional tests based on the evaluation of hydrodynamic interactions. When considering nanofilaments as a long semiflexible chain and observing its random deformation, it is possible to obtain the so-called “persistence length”, i.e. a measure of the chain’s stiffness or flexibility. The stiffer the chain, the larger the persistence length [19]. Such an approach is well known for DNA and long-molecules characterisation. Thus, we demonstrated, as described below, the ability of the optical tracking method and hydrodynamic interactions as tools for the evaluation of the mechanical properties of our flexible nanofilaments. The results are compared with estimates obtained by nanoindention performed using an atomic force microscope.

In general, our study should help in understanding both the behaviour of flexible nanofilaments conveyed by a flow, by making it possible to verify the existing theoretical models [20, 21], and the physical phenomena responsible for the folding and bending dynamics of long molecular objects.

## Materials and Methods

### Materials

Poly(L-lactide-*co*-caprolactone) (PLCL, 70% L-lactyde and 30% caprolactone unit, Corbion Purac, Netherlands), chloroform (CHCl_3_, POCh, Poland), N,N-dimethylformamide (DMF, POCh, Poland), Bovine Serum Albumin conjugated with fluoresceine (BSA-FITC, Sigma Aldrich, Poland), acrylamide (AAm, Sigma Aldrich, Poland), N,N-isopropylacrylamide (NIPAAm, 97%, Sigma Aldrich, Poland), N,N’-methylene bisacrylamide (BIS-AAm, 99.5%, Sigma Aldrich, Poland), ammonium persulfate (APS, 98%, Sigma Aldrich, Poland), N,N,N’,N’-tetramethylethylenediamine (TEMED, 99%, Sigma Aldrich, Poland). Before polymerisation of PNIPAAm gel, the NIPAAm monomer was recrystallised twice by using benzene-hexane in a ratio of 9:1 (v/v) and highly purified water obtained from a Hydrolab/HLP purification system with conductivity 0.056 μS/cm. This process was necessary for the removal of any contamination that might prevent polymerisation.

### Preparation of hydrogel nanofilaments

The electrospinning shell solution was prepared by dissolving 1 g of PLCL polymer in 10 g of mixture of DMF and CHCl_3_ 1:9 (w/w), and left for 24 hours. Preparation of a core solution of 10 wt.% AAm/BIS-AAm or NIPAAm/BIS-AAm: 2.24 g of AAm (or NIPAAm) and 0.06 g of BIS-AAm were dissolved in 20.7 g of deionised water. Mass ratio of AAm (or NIPAAm) to BIS-AAm was 37.5:1. Before electrospinning, 10 μl of APS and 0.007 g of BSA-FITC were mixed with 1 g of AAm/BIS-AAm solution. The addition of a small amount of BSA-FITC made it possible to use fluorescence for image acquisition and to study protein release dynamics [5]. Finally, a suitable amount of 1 μl of TEMED was added to the solution of AAm/BIS-AAm/APS which was then vigorously shaken. The addition of TEMED triggered hydrogel polymerisation.

The core solutions of AAm/BIS-AAm/APS/TEMED/BSA-FITC with mass ratio of AAm/BIS-AAm (w/w): 37.5:1, 20:1 and 4:1 were labelled as EA1, EA2, and EA3, respectively. The core solutions of NIPAAm/BIS-AAm/APS/TEMED/BSA-FITC with mass ratio of NIPAAm/BIS-AAm (w/w): 37.5:1, 20:1 and 4:1 were labelled as EN1, EN2, and EN3, respectively. By changing the mass ratio from AAm (or NIPAAm) to BIS-AAm, a change in nanofilament flexibility was designed, increasing filament stiffness. The PLCL solution was used as the nanofibres shell in all experiments.

Nanofibres were electrospun using a core-shell vertical setup with the outer layer flow rate (9 wt.% PLCL solution) set to Q_shell_ = 1500 μl/h. The core solution flow rate was set to Q_core_ = 1500 μl/h. Electrospinning was performed at a positive voltage of V = 15 kV. Nanofibres were collected on the microscope slide and on a rotating drum (2000 rpm) with a 3 cm diameter covered with grounded aluminium foil. Temperature and humidity during electrospinning were T = 25°C and h = 45%, respectively.

To extract hydrogels from the core-shell structure, 100 μl DMF droplet was placed on a glass slide covered with deposited nanofibres and left for 30 seconds. Then the droplet was transferred to a glass vial. This procedure was repeated to obtain a larger volume of nanofilament suspension. Afterwards, the solution was mixed with pure DMF to lower the nanofilament concentration.

**Fig 1:**
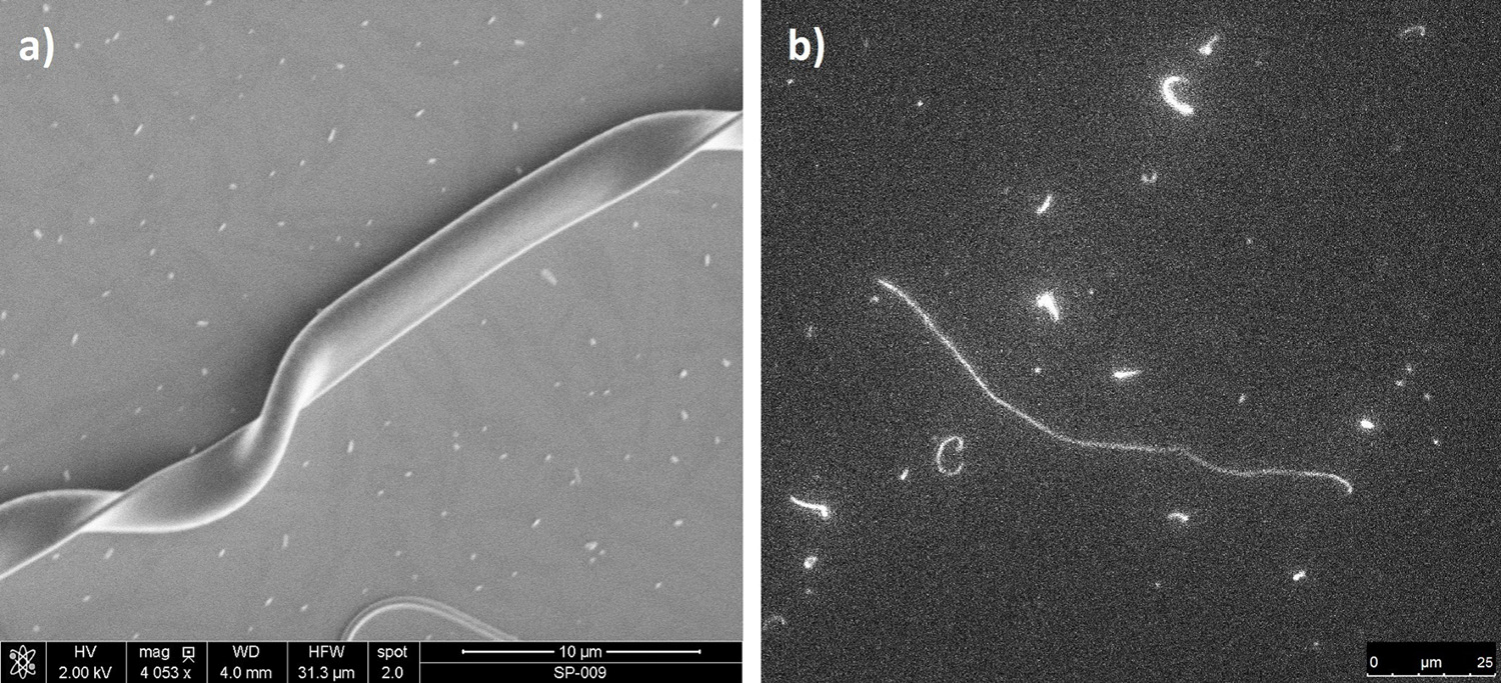
a) SEM micrograph presenting ribbon-like core-shell PLCL/PAAm nanofibre (EA3) (see file S1 Fig). b) Fluorescent nanofilaments extracted as core from nanofibre. The contour length of the longest nanofilament equals 104 μm.

Initially, several experimental trials were conducted to determine the optimal amount of TEMED to be added to the monomer solution to start polymerisation. Experiments with AAm result in 1 μl of TEMED added to 1 g of AAm/BIS-AAm/APS solution, with a polymerisation time of about 35 minutes. Polymerisation time was indicated by the clogging of the core needle during co-electrospinning. For the same quantities of reagents, NIPAAm’s polymerisation time was found to be very similar. To confirm polymerisation of the core structure, a DMF droplet was placed on the nanofibres electrospun on the microscope cover slide and observed under the fluorescence microscope (Leica DMI3000B). A wide range of nanofilaments, with contour lengths from 1 to over 500 μm and diameter well below 500 nm, was observed (Fig 1).

### Morphological characterisation

The morphology of the nanofibre mats and hydrogel nanofilaments was examined by scanning electron microscopy, using a model Nova NanoSEM 450 (FEI, USA). The mats were sputtered with gold using a model SC7620 Polaron mini sputter coater (Quorum Technologies Ltd., Ashford, UK). The average diameter of nanofibres was calculated for 100 manually selected nanofibres by using ImageJ [22] software. In the case of hydrogel nanofilaments, the average calculation was limited to approximately 10 nanofilaments due to difficulties with transferring very delicate nanofilaments onto glass cover slides as well as with the precipitation of PLCL polymer covering nanofilaments. The surface topography of samples was collected by AFM (Ntegra, NT-MDT) equipped with a closed liquid cell and a rectangular silicon cantilever (CSG01, NT-MDT). The spring constant and mechanical resonance were in the range of 0.003–0.13 N/m and 4–17 kHz, respectively. Measurements were performed in contact mode, in aqueous solutions at 25°C.

### Protein release

Six samples of PNIPAAm material (EN1–EN3) containing BSA-FITC were analysed for a protein release study, and compared with core-shell nanofibreous material without hydrogel in its core structure. For this purpose, a nanofibrous mat of 3 cm^2^ was weighed to determine its BSA-FITC content and dissolved in 1 ml of DMF to extract nanofilaments. The amount of 2.5 ml of deionised water was added to the nanofilament solution. Afterwards, the vial was vigorously agitated, and three samples of 1 ml each, were transferred to plastic vials. 1 hour after the release of BSA-FITC from the nanofilaments, the sample was centrifuged for 10 minutes at 10,000 rpm to separate the nanofilaments from the supernatant. The supernatant was transferred to clean plastic vials. The same procedure was followed for samples after 6 and 24 hours of release.

For the release study from the nanofibrous mat, 1 cm^2^ of material was weighed and immersed in 1 ml deionised water (37°C). The supernatant fluid was exchanged with fresh water after 1, 6, and 24 hours.

300 μl of collected supernatants were placed in 96-well microplate and measured with a fluorometer (Fluoroskan AscentTM Microplate Fluorometer, Thermo Scientific, USA). The concentration of released protein was calculated by a calibration curve. Additionally, the diffusion coefficient of BSA-FITC in EN1 hydrogel (37°C) was evaluated by the Fluorescence Recovery After Photobleaching method (FRAP) using a Leica TCS SP5 microscope.

### Thermal fluctuations

A prepared suspension of hydrogel nanofilaments in DMF was placed between two sealed microscope slides. The distance between glass covers was about 50 μm. The Brownian motion of short nanofilaments was recorded using high-gain EM-CCD camera (C9100-2, Hamamatsu, Japan) coupled with a Leica AM TIRF MC epifluorescence microscope. Single nanofilaments were observed under a 63 × 0.70 NA objective lens. The sampling rate was approximately 10 frames per second with 1000 frames recorded in sequence. Matlab [23] script was developed to locate, for each analysed filament, its centroid [x(t), y(t)] in the laboratory coordinate frames, and the filament orientation Θ(t) relative to x axis in the frame. The incremental displacement of the nanofilament observed under the microscope between two time steps [δa_n_, δb_n_] was calculated in the chosen laboratory coordinates using a rotation matrix [24].

Long sequences (-1000) of images were used to obtain translational diffusion coefficients along the *a* and *b* axes, calculated using mean squared displacements for a given time interval [24] <[Δ(t)]^2^> = 2D_a_t, <[Δb(t)]^2^> = 2D_b_t. Similarly, the rotational diffusion coefficient was calculated from the mean square angular displacement <[ΔΘ(t)]^2^> = 2D_Θ_t.

Our experimental results were compared with the theoretical diffusion coefficients obtained for a long thin (L >>R) prolate spheroid [24, 25], and expressed by Eq 1.

$$\begin{array}{c} D_{a}=\frac{k_{B}T\left[ln\left(L/R\right)-0.5\right)]}{2\pi \eta_{s}L}\;\;\;\;D_{b}=\frac{k_{B}T\left[ln\left(L/R\right)+0.5\right)]}{4\pi \eta_{s}L}\;\;\;\;D_{\Theta }=\frac{3k_{B}T\left[ln\left(L/R\right)-0.5\right)]}{\pi \eta_{s}L^{3}} \end{array}$$ (1)

where k_B_ is Boltzmann’s constant, T is the absolute temperature, η_s_ is the viscosity of the water at temperature T, Θ is the rotation angle, and L, R are the length and radius of the filament.

To evaluate the mechanical properties of flexible nanofilaments we analysed their shape change due to thermal fluctuations [26–28]. With this method, the bending stiffness of polymers such as microtubules or DNA can be described by their persistence length. According to work [29], the persistence length for thermally driven fluctuations can be defined by Eq 2.

$$\begin{array}{c} L_{p}=\frac{E\;I}{k_{B}T} \end{array}$$ (2)

where *E* is the Young modulus, and *I* = πR_f_^4^/4 is the moment of inertia of the filament cross-section. In the experiment the persistence length *L_p_* was calculated from two-dimensional cosine correlation between two unit tangent vectors of the deformable filament [27, 29] according to Eq 3.

$$\begin{array}{c} \left\langle cos\theta \left(s\right)\right\rangle =e^{-L/2L_{p}} \end{array}$$ (3)

where L is the contour length of the filament, and θ is the angle between two unit tangent vectors. Brackets denote the cosine average over all the analysed positions.

For the purpose of persistence length determination, prepared hydrogel nanofilaments were observed between two closely arranged microscope slides sealed with grease to prevent convecting flow. Only long (>10 μm) nanofilaments were analysed to record fluctuations dominating in two dimensions. Input images were prepared according to the recommendations given in papers [27, 30]. Our improved version of the Matlab script based on the De La Cruz group software [30] was used for the persistence length evaluation.

### Hydrodynamic interactions

The hydrodynamic method used for Young modulus determination is based on the analysis of nanofilament bending by fluid motion [28]. For this purpose fibres were electrospun on a PDMS substrate with a hollow channel with a 5 mm inner diameter. Care was taken to obtain the perpendicular arrangement of fibres in relation to the channel length. The initiation of fluid flow in the channel caused the bending of the hydrogel nanofilament. Stopping the fluid flow brought the nanofilament to return to its original position. The evaluation of the filament’s deformation rate and hydrodynamic drag forces makes it possible to determine its elastic properties. In order to avoid uncertainties in the estimate of very low flow rates of the fluid, the elastic coefficient of the filament was determined for the returning phase of the process, when hydrodynamic drag-retarded filaments recover their initial position.

Neglecting the inertial effects, the elasticity force of the recovering filament is in equilibrium with the hydrodynamic drag force. For the creeping flow regime it can be approximated [25] by the hydrodynamic interaction of a thin ellipsoid with length *L* and radius *R* which moves in the fluid perpendicularly to its main axis with velocity *U*, according to Eq 4.

$$\begin{array}{c} F_{D}=\frac{4\cdot \pi \cdot \mu \cdot U\cdot L}{\ln(L/R)}, \end{array}$$ (4)

where μ is fluid viscosity.

### Atomic Force Microscopy nanoindentation

Nanoindentation analysis was performed to measure directly the Young modulus of hydrogel nanofilaments immersed in water. Filament topography was also compared with measurements performed in air. Stiffness measurements were carried out using AFM (Ntegra, NT-MDT) at 25°C in a closed liquid cell in order to keep the hydrogel hydrated, prevent contamination, and to minimise the influence of thermal drift. A silicon nitride cantilever (CSG01, NT-MDT) with nominal tip radius of 6 nm was used. The spring constant of the cantilever, calibrated using the thermal method, was 0.040 N/m. Selected hydrogel nanofilaments were analysed by collecting a series of force curves from several areas. In this treatment, nanofilaments were considered as cylinders and the AFM tip as a sphere [31]. The filament Young modulus was calculated by fitting the Hertz model into the loading data, using the procedure reported by Tan et al. [31].

## Results

### Hydrogel nanofilament morphology

**Fig 2:**
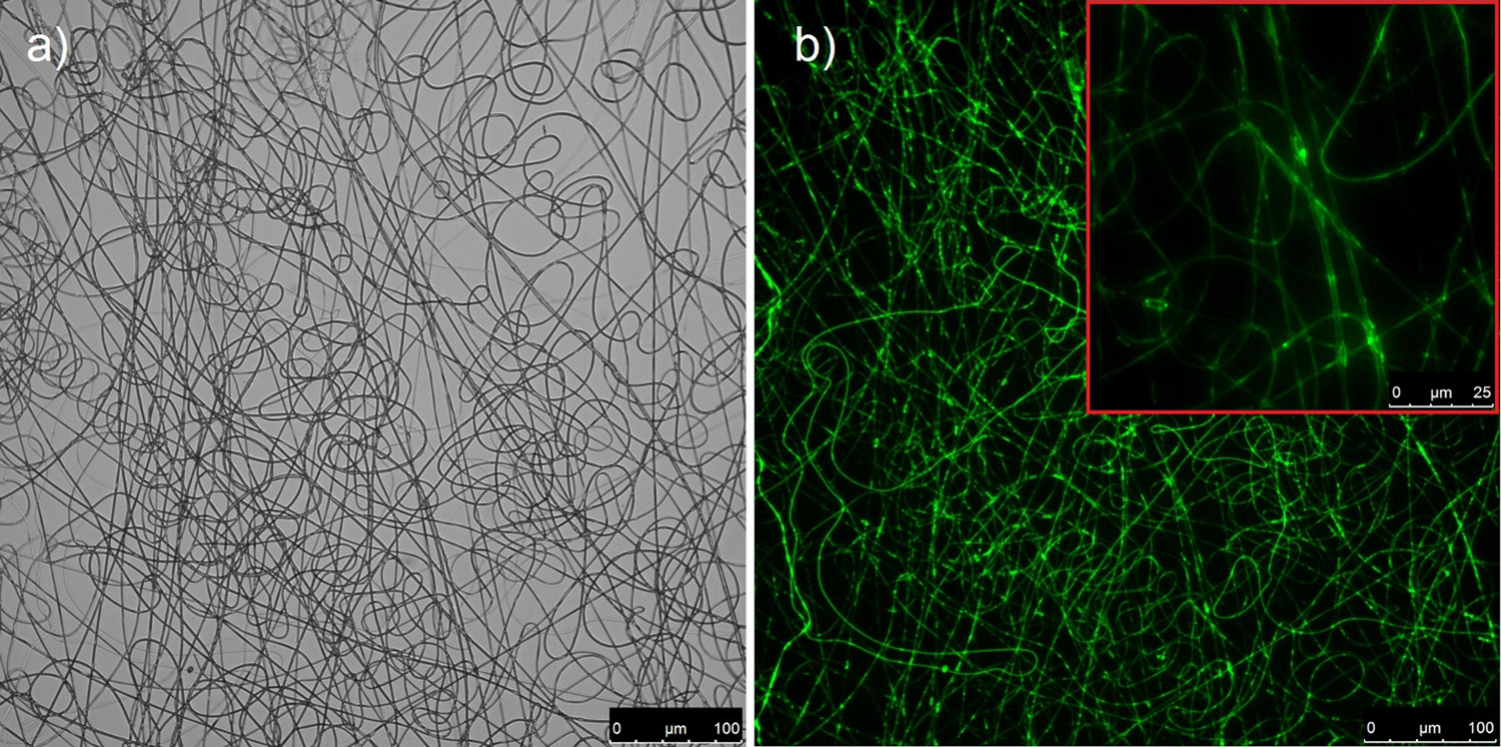
Optical (a) and fluorescence (b) micrographs of electrospun core-shell PNIPAAm/PLCL fibres. The red framed insert shows highly magnified fibres.

The fluorescence images of electrospun fibres (comp. Fig 2) confirm the presence of their core-shell structure. Fluorescent micrographs indicate continuous core segments in fibres, although after the dissolution of the PLCL shell, numerous short objects can also be found in addition to the long hydrogel nanofilaments. Magnification in the Fig 2b shows the presence of a narrowing over the length of the fibre, appearing as a lower fluorescence intensity in these regions. Such nonuniformities may influence nanofilament bending dynamics. Average diameters obtained from the SEM images of nanofibers from materials EN1, EN2, and EN3 were 1.35 ± 0.38 μm, 0.71 ± 0.31 μm, and 0.79 ± 0.15 μm, respectively. Similarly, the average diameters of nanofibers from materials EA1, EA2 and EA3 were 1.98 ± 0.21 μm, 2.1 ± 0.45 μm, and 1.27 ± 0.18 μm, respectively. No differences between the microstructure of PLCL/PAAm or PLCL/PNIPAAm nanofibres were visible under the fluorescence microscope or from SEM micrographs.

In the case of materials EA2, EA3 and EN2, EN3, polymerisation time strongly decreased. For experiments EA3 and EN3, polymerisation took place approximately one minute after the addition of TEMED, practically blocking the spinneret needle before electrospinning started. In order to electrospin these materials, the core solution was electrospun without TEMED. The collected nanofilaments were found, nonetheless, to be polymerised. Most likely, the charge motion due to the high voltage applied during electrospinning of polymer solutions initiated free radical production, and thus the polymerisation of AAm:BIS-AAm or NIPAAm:BIS-AAm monomers.

**Fig 3:**
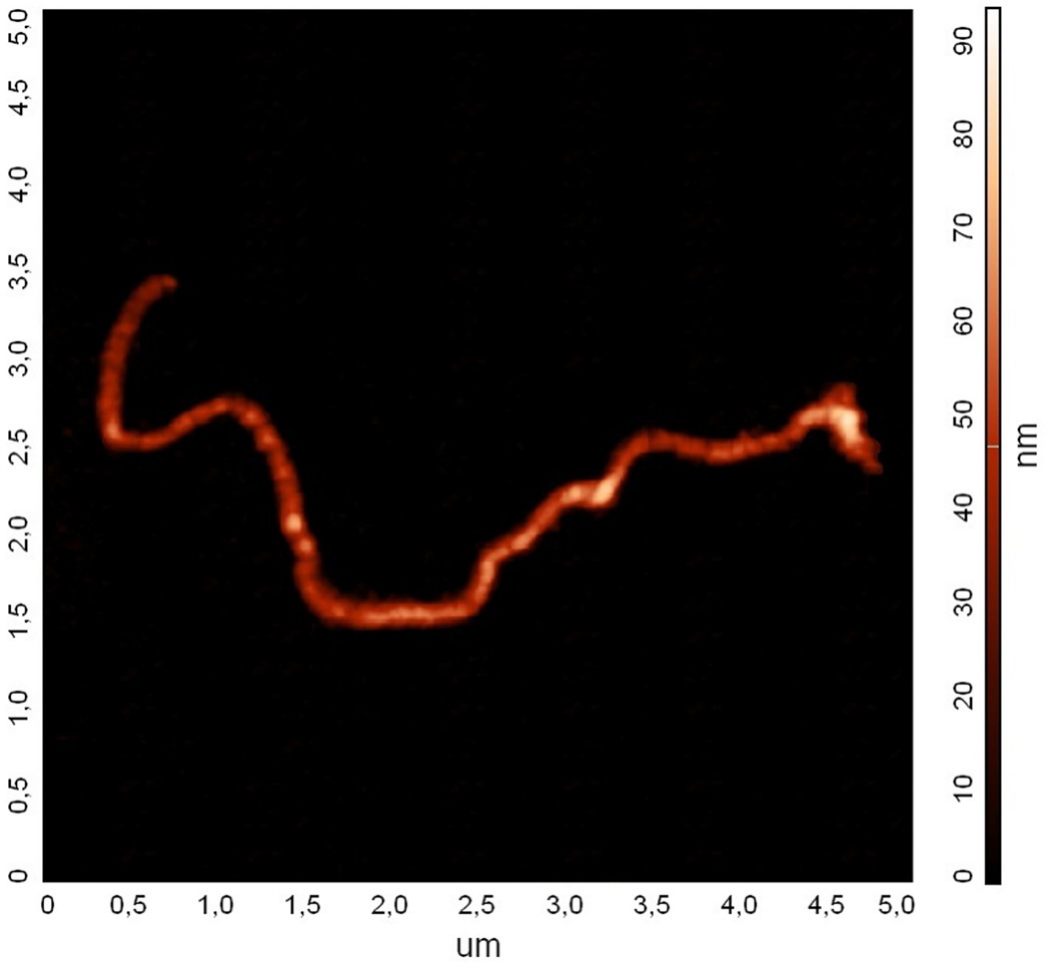
Ribbon-like nanofilament (EA1) observed under an atomic force microscope; contour length 7 μm, width 128 nm, height 39 nm; equivalent radius R_f_ = 40 nm. (see file S2 Fig).

AFM topographies of hydrogel nanofilaments obtained after PLCL shell dissolution were collected in order to evaluate their average diameter. For PNIPAAm hydrogel nanofilaments from materials EN1, EN2, and EN3 the average diameters were 126 ± 36 nm, 170 ± 65 nm, and 257 ± 207 nm, respectively. Similarly, the average diameter of PAAm hydrogel nanofilaments from materials EA1, EA2, and EA3 were 96 ± 24 nm, 112 ± 47 nm, and 290 ± 209 nm, respectively. Fig 3 shows an AFM image of a typical nanofilament adhering to a glass surface. The diameters of nanofilaments obtained by the AFM technique was comparable with those obtained from SEM images.

### Release study

**Fig 4:**
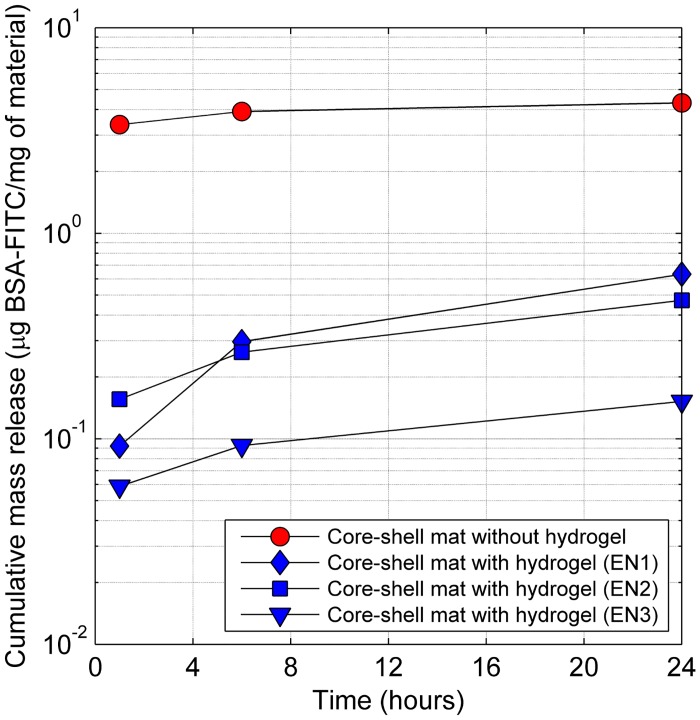
Cumulative release of BSA-FITC from core-shell mat with NIPA hydrogel in the core, and core-shell mat with protein water solution in the core. Standard deviation was less than 5%. For better visibility of the results, the *y* axis is in a logarithmic scale.

**Fig 5:**
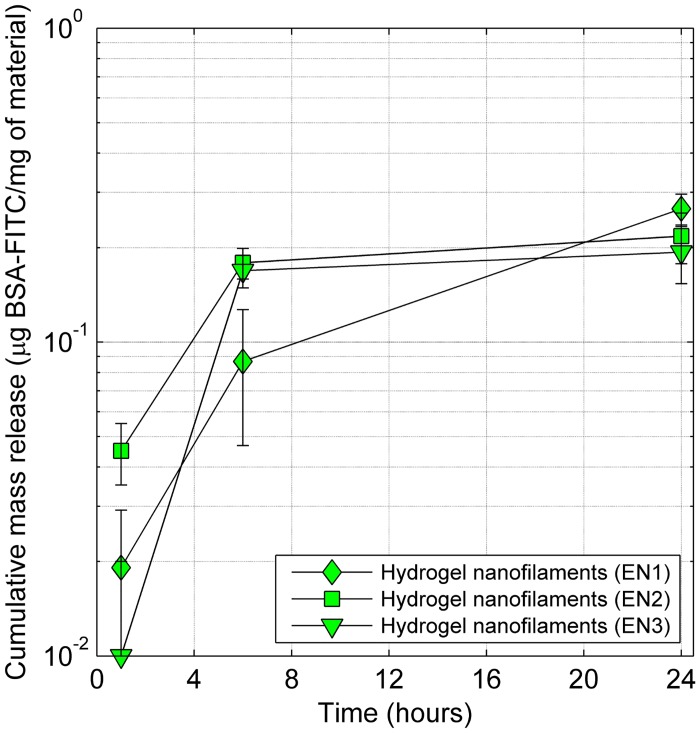
Cumulative release of BSA-FITC from hydrogel nanofilaments of three different compositions. For better visibility of the results, the *y* axis is in a logarithmic scale.

Release kinetics of BSA-FITC hydrogel material trapped in the core of nanofibers are shown in Fig 4 and release from extracted nanofilaments are shown in Fig 5. These characteristics are compared with simple nanofibrous material with BSA-FITC, which was previously used for drug release modelling [5]. Worthy of note is the substantial decrease in the proteins released from extracted nanofilaments as compared to those still entrapped in the core of nanofibers. This may be attributed to the precipitation of shell polymer after the addition of water to dissolved materials as well as to the absorption of nanofilaments, which thus prevent access to the fluid. The release profile of protein from the material without hydrogel in the core structure was almost seven times higher after 24 hours, when compared to the material with PNIPAAm hydrogel in the core (EN1). This value is in agreement with our previous observations [5, 32]. The protein release kinetics for materials of different compositions shows the highest release after 24 hours for nanofibrous mat with the lowest degree of cross-linking of the hydrogel (EN1) (Fig 4). Similarly, nanofilaments with different composition shows the highest release after 24 hours for EN1 nanofilaments (Fig 5). The absence of hydrogel in the core led to burst release, probably caused by a leakage of protein solution through nano ruptures in the nanofibre shell. The diffusion coefficient of BSA-FITC in hydrogel EN1 (D_BSA-FITC_ = 7.3 ± 3.7 μm^2^/s) is three orders of magnitude lower than the diffusion coefficient in water, therefore the release from new hydrogel materials can be significantly prolonged.

**Table 1:** Comparison between experimental and theoretical diffusion coefficients for selected deformable nanofilaments of type EN1. Diffusion measured between two cover slides about 50 μm apart. Theoretical values for the ideal nondeformable ellipsoid were calculated from Eq (1). Persistence length L_p_ obtained by cosine correlation. Measurements performed in water at temperature T = 30°C, viscosity η_s_ = 1.02⋅10^-3^ Pa s.

**Nr**		**L/2R**	**D_a_(μm^2^)**	**D_b_(μm^2^)**	**D_Θ_ (rad^2^)**	**L (μm)**	**L_p_**	**Motion**
**1**	exper.	43.0	0.052	0.035	0.0006	21.5	93	Bending
	theor.	43.0	0.144	0.090	0.0019			
**2**	exper.	31.2	0.093	0.067	0.0025	15.6	29	Rotation
	theor.	31.2	0.182	0.116	0.0045			
**3**	exper.	29.8	0.059	0.140	0.0029	14.9	339	Rotation
	theor.	29.8	0.189	0.121	0.0051			
**4**	exper.	47.8	0.231	0.053	0.0005	23.9	211	Bending
	theor.	47.8	0.133	0.083	0.0014			
**5**	exper.	31.4	0.266	0.070	0.0023	15.6	598	Rotation
	theor.	31.4	0.182	0.116	0.0044			
**6**	exper.	48.4	0.112	0.105	0.0026	24.2	5	Bending
	theor.	48.4	0.132	0.082	0.0013			
**7**	exper.	104.8	0.054	0.070	0.0005	52.4	5	Bending
	theor.	104.8	0.072	0.044	0.0002			
**8**	exper.	171.0	0.039	0.150	0.00005	85.5	31	Bending
	theor.	171.0	0.049	0.029	0.00004			
**9**	exper.	105.6	0.029	0.087	0.0002	52.8	15	Bending
	theor.	105.6	0.072	0.043	0.0002			

### Mechanical properties of hydrogel nanofilaments

#### Thermal fluctuations

The diffusion coefficients calculated from the mean square displacements of the suspended nanofilaments are presented in Table 1. It can be seen that, for shorter nanofilaments (Nr 1–5, Table 1), the diffusion coefficients measured by tracking analysis are on average two times smaller than the theoretical ones for the oblate ellipsoid (Eq 1). For longer nanofilaments (Nr 6–9, Table 1), the opposite is true. On average, diffusion coefficients from experimental analyses are almost two times higher than in theory. It is worth remembering that the theoretical values are obtained by using the radius and length of the observed filaments as the main axis of a virtual ellipsoid. Therefore, the theoretical values of the diffusion coefficients must be taken for what they are, only as a reference in this very simplified model.

**Fig 6:**
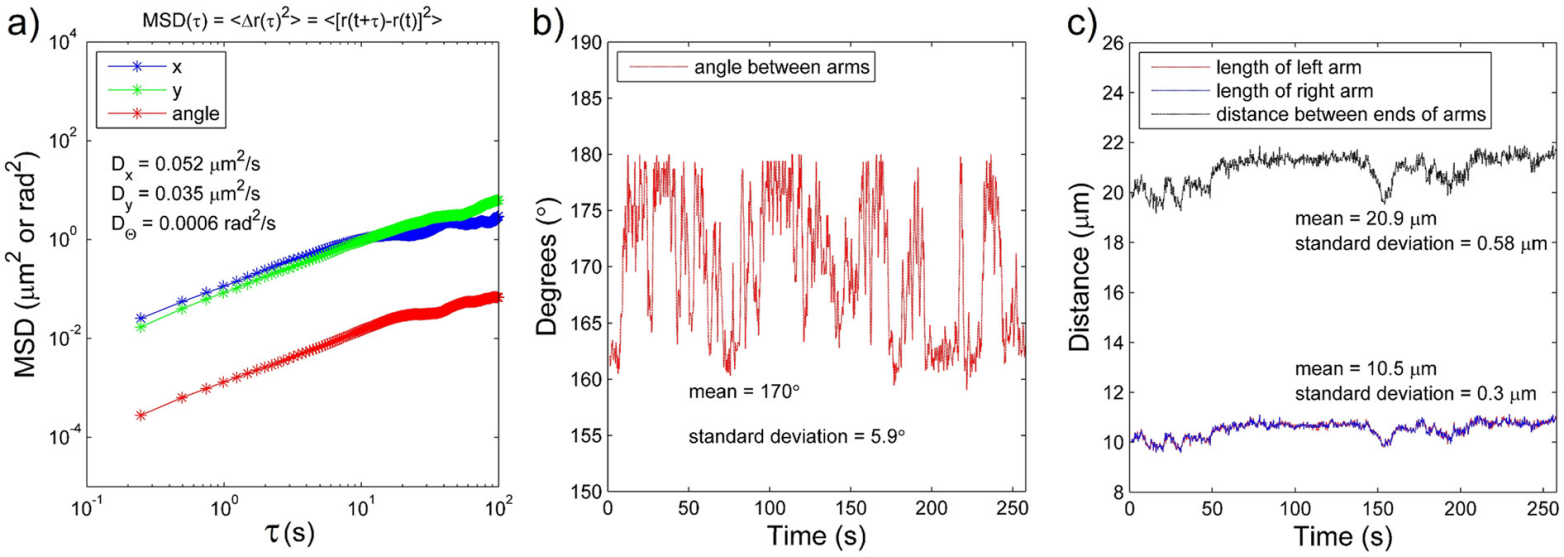
Fluorescence images showing bending dynamics of a nanofilament (Table 1, nanofilament no. 1). Red lines indicate arms of the fibre starting from the centre of the fibre mass. The angle between the red lines was measured to assess flexibility. The time interval between frames is t = 0.25 s.

**Fig 7:**
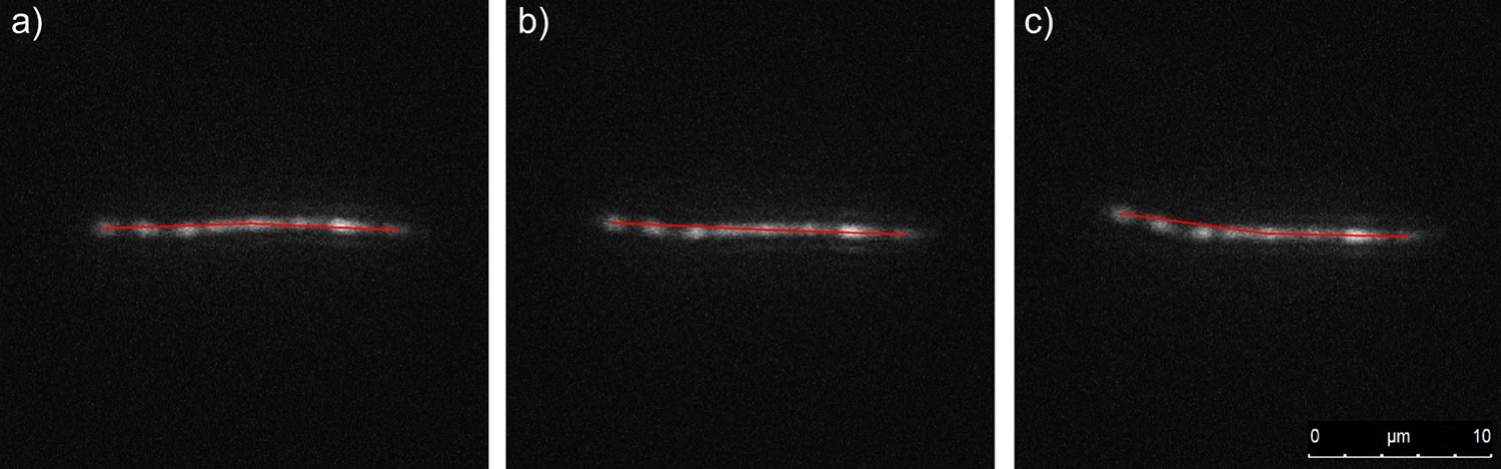
a) Plot of the mean square displacement of a filament of contour length 21.5 μm as a function of lag time. The upper two plots are MSDs along the *a* and *b* axes in terms of μm^2^, whereas the bottom one is the angular MSD in terms of mrad^2^. b) Angle between arms of the bending filament as a function of time. c) Length of left and right arm of the bending filament, and distance between both ends of the arms. All plots present calculations for the nanofilament No. 1 from Table 1.

In the first nanofilament we can observe multiple narrowings (Fig 6), which probably influenced bending dynamics due to thermal fluctuations. Fig 7 shows the mean square displacement of the above-mentioned nanofilament, which was evaluated during 100 second experimental time for the translational diffusion along the *a* and *b* axes as well as for the rotational displacement. The curves obtained are approximately linear. Given the fact that nanofilaments might bend during examination, we divided the analysed object into two segments and tracked three main points of the nanofilament: its centre and both ends. By analysing the angle between two arms of the nanofilament we were able to assess bending dynamics due to thermal fluctuations (Fig 6b). The nanofilament arm span varied in the range of 11.8°. In addition, in order to confirm the bending in contrast to rotation, the length of the arms and distance between both ends of the arms was measured. For C-shaped nanofilaments, the possible rotation of the nanofilament would cause its ends to move out of focus and apparently decrease their projected length. This effect was used to rule out such cases during bending evaluation. As we may see in the presented data (Fig 6c), the variation in both the arm lengths and the distance between the ends is only about 3%, thus indicating a dominant bending motion.

**Fig 8:**
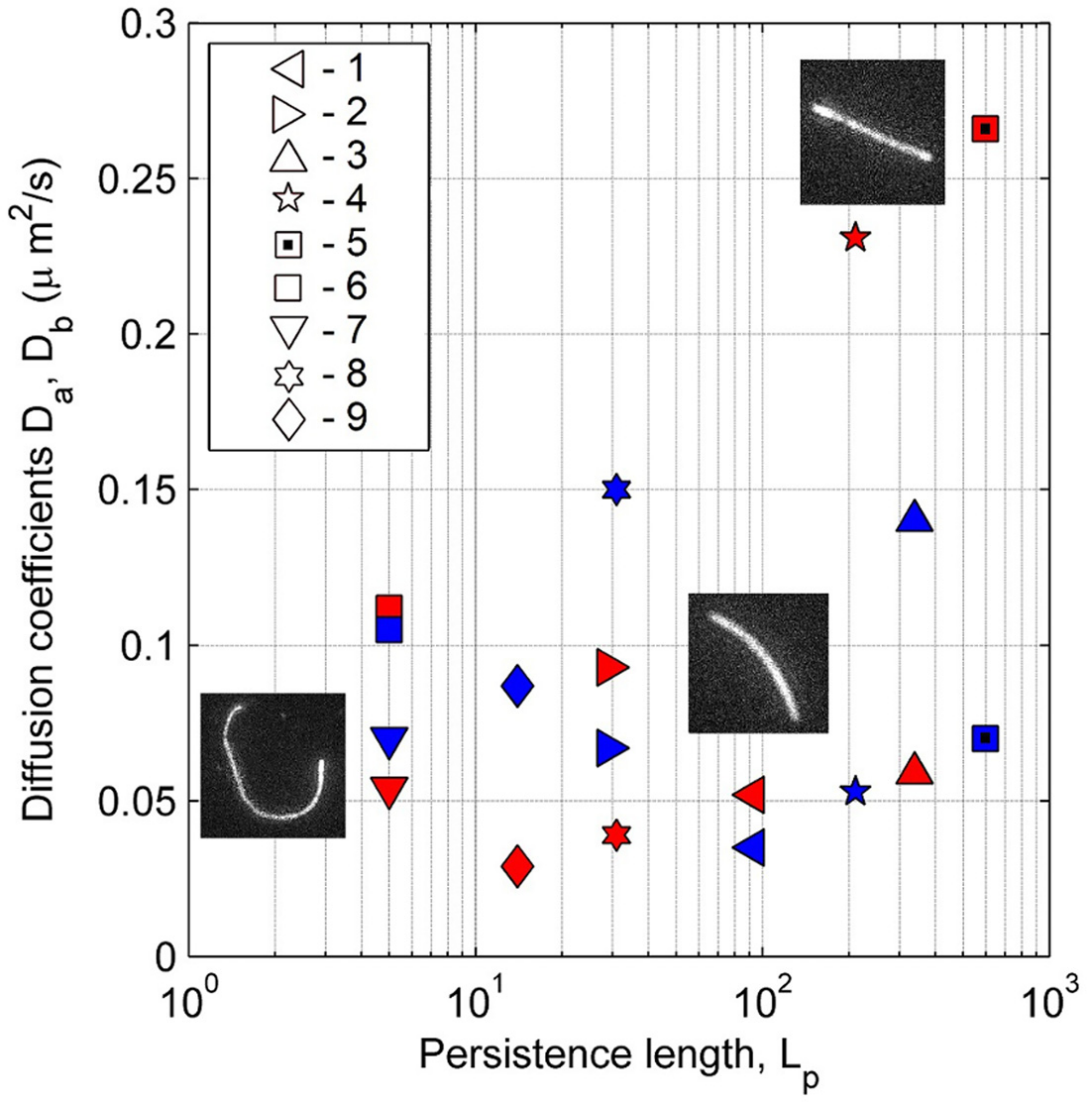
Diffusion coefficient D_a_ (red symbols) and D_b_ (blue symbols) as a function of persistence length for nine analysed filaments. Each symbol number represents data from an appropriate line of Table 1.

Young modulus values measured by the persistence length with the cosine correlation method for nanofilaments of different monomer ratios are gathered together in Table 2. It is worth noting that in several cases filaments exhibited a partial narrowing of their diameter and/or had a ribbon shape (Figs 1b and 3). An example of the bending dynamics for such nanofilaments is presented in Fig 6. The sequence of images obtained at the time interval of 0.25 s shows the partial bending in thinner parts of the nanofilament; however, its thicker parts apparently have a stiffer behaviour and their bending is less visible. Data presented in Table 2 for the cosine correlation method did not show an increasing trend of Young modulus with increased cross-linker amounts as was found in the case of AFM indentation. It can be seen that nanofilaments from N,N-isopropylacrylamide (PNIPAAm) immersed in water are slightly more flexible than acrylamide (PAAm) filaments: an aspect consistent with the results found in literature. Fig 8 combines data obtained for persistence length with two longitudinal diffusion coefficients of nine evaluated nanofilaments. In general we may note very weak relations between flexibility (persistence length) and diffusion. In two cases (4, 5) relatively high longitudinal diffusion is probably associated with higher stiffness and a straight filament shape. This indicates the need to include shape parametrisation when seeking a possible correlation between diffusion and persistence length.

When comparing the performance of our own script with the Persistence software [30], we found its greater flexibility when fitting with Eq (3). More reliable results were obtained for images of apparently segmented objects. In any case, it seems that quantitative Young modulus measurements based on the persistence length need a more detailed evaluation of the filament shape, especially in order to analyse cases where the non-uniformity of filaments and their ribbon-like shape dominate.

#### Hydrodynamic interactions

For the calculation of a Young modulus from the bent filament (comp. Table 2) we used data for a time step of three seconds and five seconds after the flow stopped. The analysed filament consisted of a rigid, rod-like part of length L_1_ = 145.38 μm, and a flexible part of length L_2_ = 39.14 μm attached to the channel wall. The observed rigid part was moving to the original position with an apparently constant angular speed. The measured linear velocity *U* at any point on the filament was 1.34 μm/s. Thus the drag force acting on a stiff fibre part according to Eq (4) equals 4.53 ⋅10^-13^ N. The resulting Young modulus calculated for the flexible part of the filament equals 2.34 kPa. This value is comparable to that obtained by the persistence length method. Due to complications in finding filaments attached perpendicularly to the channel wall, only one experimental case (EN1) was evaluated by this method.

#### Atomic Force Microscopy nanoindentation

In Table 2, several AFM measurements of a Young modulus are given for hydrogel filaments. The atomic force microscopy indentation method has limited applicability for highly deformable objects, mainly due to the relatively high stiffness of the cantilever as compared to the fluid-like behaviour of hydrogel. The Young modulus value is obtained from the slope of the loading curves and by averaging the results obtained from the calculation of all the recorded measurements for each sample. In most cases it is in the area of several kPa, generally in agreement with available data [33]. However, we were unable to obtain reliable data for the highly deformable gel labelled as EN1 (Table 2). The scatter of obtained data is relatively high due to the tedious procedure of catching the proper part of a filament, their non-uniform geometry, and filament interaction with the environment. Thus the mechanical properties measured *in situ* by using an atomic force microscope may deviate greatly from those exhibited by the filaments suspended in fluid.

**Table 2:** Young modulus from AFM indentation, cosine correlation method, and filament bending in the flow. Three types of nanofilaments with different NIPAAm:BIS-AAm and AAm:BIS-AAm mass ratios were used. Experiments EN3 and EA3 were carried out without TEMED initiator; nevertheless polymerisation occurred *“in flight”*, during electrospinning. For AFM indentation the average Young modulus obtained for three different nanofilaments is given; for the cosine correlation method about 500–700 images per nanofilament were analysed using Eq (3), formulated by Ott et al. [27]. For the measurement of a Young modulus in flow, only one filament was used.

Experiment	Young modulus E (kPa)		
	AFM indentation	Cosine correlation	Flow
EN1	—	4.5±0.4	2.3
EN2	8.50±1.19	3.1±1.7	—
EN3	18.11±4.85	3.8±1.1	—
EA1	4.06±1.18	6.1±2.6	—
EA2	15.80±2.77	5.0±1.1	—
EA3	55.82±5.64	5.8±0.8	—

## Discussion

We demonstrated the possibility to produce new nanoscale materials with a potentially broad range of biological applications. Prepared protein delivery systems containing a hydrogel core showed a slower release and absence of burst release when compared to materials without hydrogel. What is more, the change in polymer concentration and monomers ratio in hydrogel, affecting diffusion coefficient, will make it possible to adapt the release profile to the medical treatment conditions. The present study, aimed to show protein release kinetics alteration caused by the presence of the hydrogel in the nanofibres core was the preliminary study of more complex and expensive study with the use of anti-cancer drugs like doxorubicin. We hope that it is valuable information for future development of thermo-responsive hydrogel based drug release vehicles made of nanofibrous structures which can act as a reversible on-off switching release systems.

Insofar as we could see, the evaluation of the Brownian motion experiments conducted offers a relatively simple way to deduce the persistence length of very soft, flexible objects. However, the quantitative determination of some mechanical properties strongly depends on the accurate measurement of the diameter of the filament. The resolution of optical measurements is limited by the light wavelength; therefore, additional techniques must be applied in order to obtain an accurate nanofilament geometry. At the present stage of our study, the radius of immersed filaments was determined by using fluorescence microscopy, and only partly validated by AFM or SEM measurements. In most cases it was impossible to carry out such double checking for exactly the same filament.

The same limitation applies for the determination of Young modulus of prepared nanofilaments using the thermal fluctuation method. In Eq (2), the moment of inertia of the filament cross-section depends on the radius to the power of four. On the other hand, the experimental control of the nanofilament diameter is rather difficult. It might be achieved by changing the monomer ratio. We observed an increase in the filament diameter as the BIS-AAm amount increased. This effect was perhaps induced by the polymerisation of the core solution already during electrospinning, rather than post-electrospinning on the collector. While electrospinning, the polymerised core prevented a further elongation of the fibre, causing subsidence of the thick nanofibres on the collector.

The images obtained by AFM frequently indicate a ribbon-like shape of analysed nanofilaments. The ratio of height to width varied from about 5 to over 20. This variation seems to be correlated with the cross-linker concentration. It is likely that a faster polymerisation did not leave enough time for the nanofibre shape relaxation after it issued from the electrospinning nozzle [34]. Furthermore, it is worth mentioning that the typical height of such filaments observed under the atomic force microscope is quite small, in the range off 30 nm to 60 nm. Thus the dominant bending direction is most probably perpendicular to the filament’s smallest dimensions. This should be encountered in evaluation formulas. In all our present evaluations we assumed uniform filaments with a circular cross-section and used an area-equivalent radius; therefore, considerable deviations in the evaluated results may be expected due to anisotropic mechanical deformation.

The elasticity value obtained by the hydrodynamic interaction method was evaluated for a relatively thick filament (R = 1 μm). Its size was estimated from microscope images, thus with accuracy limited by the optical resolution. The proper estimation of the diameter is crucial for the elasticity evaluation. We must remember, too, that the hydrodynamic method presented here is also plagued by uncertainty in the fixed-wall mechanics.

The inability to accurately determine the diameter of the analysed objects, obviously caused some serious errors in our preliminary study on the mechanical properties of hydrogel nanofilaments. Shape nonuniformity caused an additional complication for the quantitative theoretical analysis of their flow-induced deformations. On the other hand, a nonuniform shape is typical for most biological objects (proteins, DNA); therefore, for example, the evaluation of ribbon-like filament mechanics may offer additional advantages for future theoretical modelling. The actual experimental issue to be solved is that of finding some methods for the precise control of the geometry of the filaments produced.

## Conclusions

Our hydrogel nanofilaments extracted from a core-shell structure are highly flexible and may find possible use as carriers and/or indicators in biomedical applications. Hydrogels are known to be biocompatible and non-irritant by nature. Therefore a vast area for their biomedical applications exists, e.g. as environment-sensitive drug delivery systems and in various types of pharmaceutical products [1, 2]. Their mechanical behaviour resembles that of long biological molecules. We showed that our flexible nanofilaments are suitable for studying the Brownian motion, bending, and folding dynamics of long deformable objects. From the present experiments we found that the diffusion coefficients obtained for such objects generally differ from the theoretical predictions based on a drag force for stiff ellipsoidal nanorod. This obviously indicates the need for a more sophisticated approach considering long, deformable filaments in models. The majority of existing models describing the dynamics of flexible objects, such as DNA or long molecules, use the simple approximation of interconnected identical spherical beads [20]. The incorporation of models of filaments with variable diameter and stiffness is a challenging task for future theoreticians.

## Supporting Information

S1 Fig AFM topography presenting core nanofilaments of different composition. a) EA1, b) EA2, c) EA3, d) EN1, e) EN2, f) EN3.
(DOCX)

S2 Fig SEM micrographs presenting core-shell nanofibers of different composition. a) EA1, b) EA2, c) EA3, d) EN1, e) EN2, f) EN3.
(DOCX)
