# Correction: Hydrogel Nanofilaments via Core-Shell Electrospinning

**DOI:** 10.1371/journal.pone.0133458

**Published:** 2015-07-20

**Authors:** Paweł Nakielski, Sylwia Pawłowska, Filippo Pierini, Wioletta Liwińska, Patryk Hejduk, Krzysztof Zembrzycki, Ewelina Zabost, Tomasz A. Kowalewski

The images for Figs 6 and 7 are incorrectly switched. The image that appears as Fig 6 should be Fig 7 and the image that appears as Fig 7 should be Fig 6. The figure captions appear in the correct order. Please view the correct figures below.

**Fig 6:**
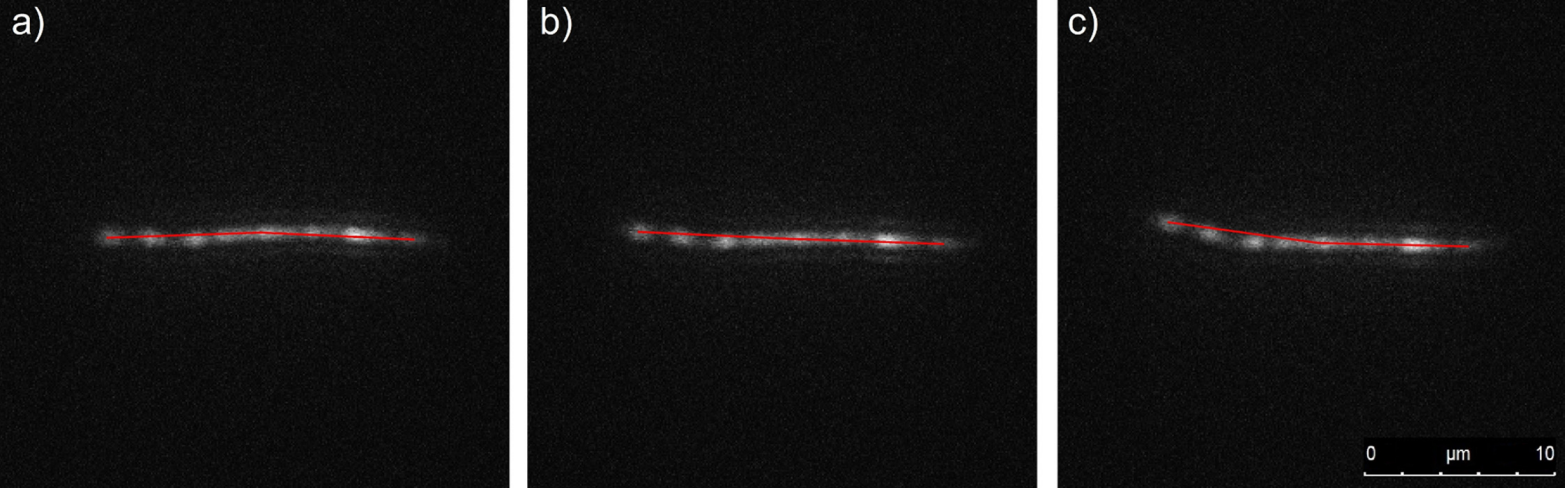
Fluorescence images showing bending dynamics of a nanofilament (Table 1, nanofilament no. 1). Red lines indicate arms of the fibre starting from the centre of the fibre mass. The angle between the red lines was measured to assess flexibility. The time interval between frames is t = 0.25 s.

**Fig 7:**
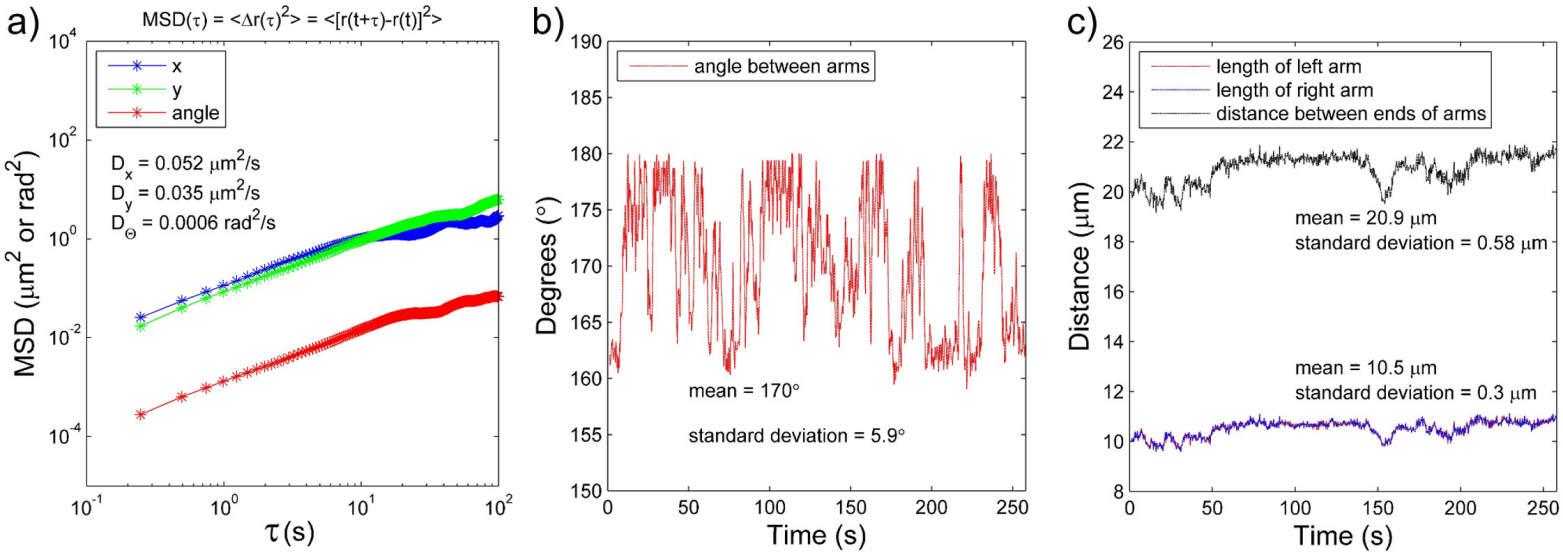
a) Plot of the mean square displacement of a filament of contour length 21.5 μm as a function of lag time. The upper two plots are MSDs along the *a* and *b* axes in terms of μm^2^, whereas the bottom one is the angular MSD in terms of mrad^2^. b) Angle between arms of the bending filament as a function of time. c) Length of left and right arm of the bending filament, and distance between both ends of the arms. All plots present calculations for the nanofilament No. 1 from Table 1.

The fifth sentence in the second paragraph of the Results subsection titled “Mechanical properties of hydrogel nanofilaments” should reference Fig 7b instead of Fig 6b.

The tenth sentence in the second paragraph of the Results subsection titled “Mechanical properties of hydrogel nanofilaments” should reference Fig 7c instead of Fig 6c.
