# Supplementary material for: Hydrogel Nanofilaments via Core-Shell Electrospinning
Source: PLoS One. 2015 Jun 19;10(6):e0129816. doi: 10.1371/journal.pone.0129816 (PMC4474634; doi:10.1371/journal.pone.0129816)
Supplement: S1 Fig — a) EA1, b) EA2, c) EA3, d) EN1, e) EN2, f) EN3. (DOCX) [file pone.0129816.s001.docx]

| **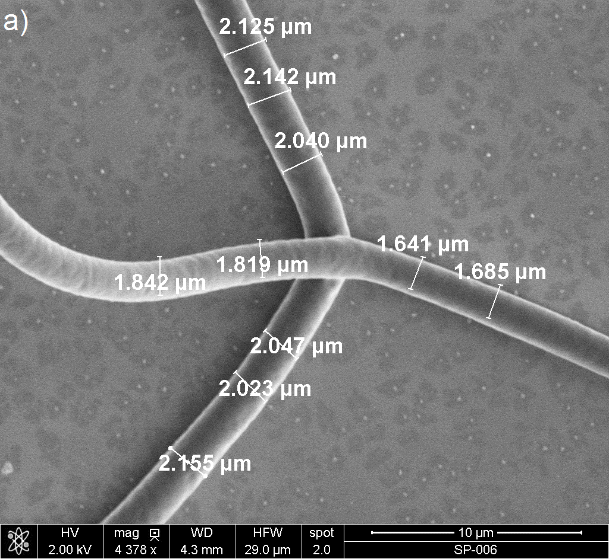** | **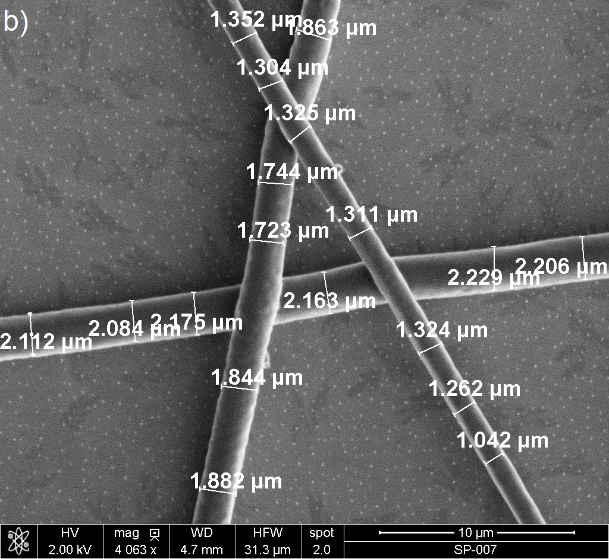** |
| --- | --- |
| **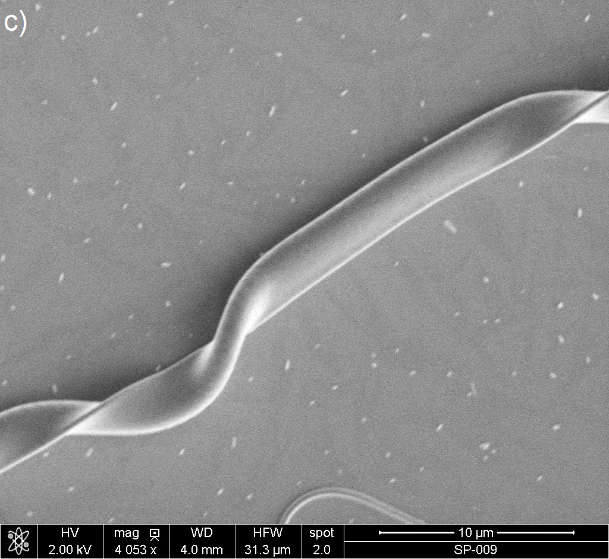** | **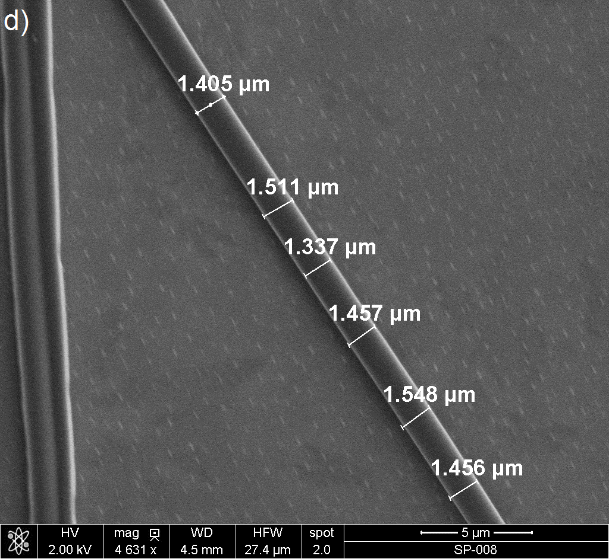** |
| **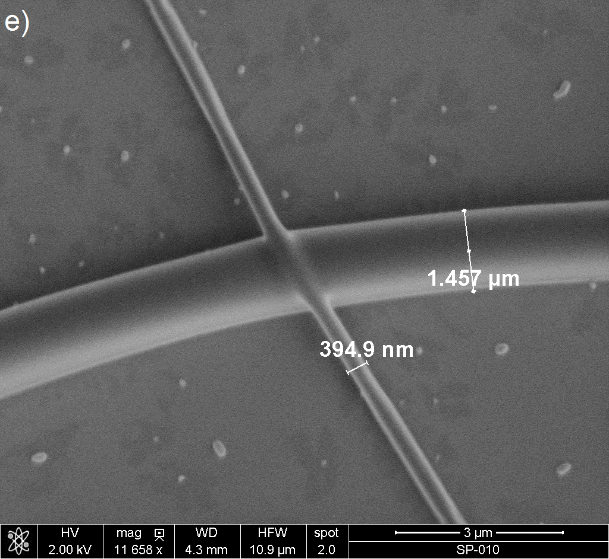** | **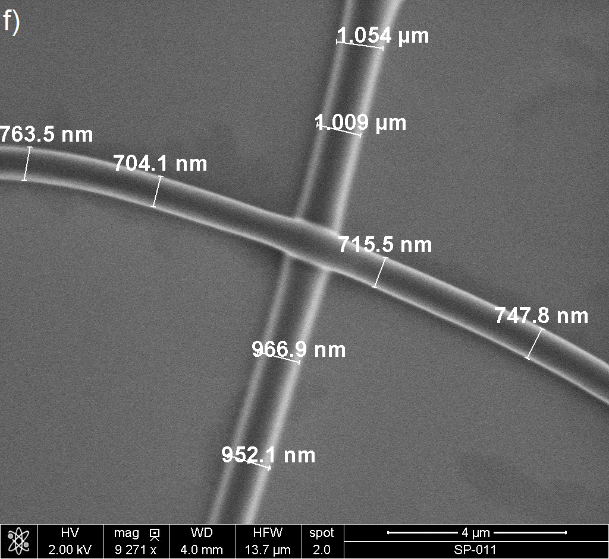** |
| **S1 Fig.** SEM micrographs presenting core-shell nanofibers of different composition: **a)** EA1, **b)** EA2, **c)** EA3, **d)** EN1, **e)** EN2, **f)** EN3. | |

**SUPPORTING INFORMATION for Hydrogel nanofilaments via core-shell electrospinning**
