# Supplementary material for: Hydrogel Nanofilaments via Core-Shell Electrospinning
Source: PLoS One. 2015 Jun 19;10(6):e0129816. doi: 10.1371/journal.pone.0129816 (PMC4474634; doi:10.1371/journal.pone.0129816)
Supplement: S2 Fig — a) EA1, b) EA2, c) EA3, d) EN1, e) EN2, f) EN3. (DOCX) [file pone.0129816.s002.docx]

**SUPPORTING INFORMATION for Hydrogel nanofilaments via core-shell electrospinning**

| 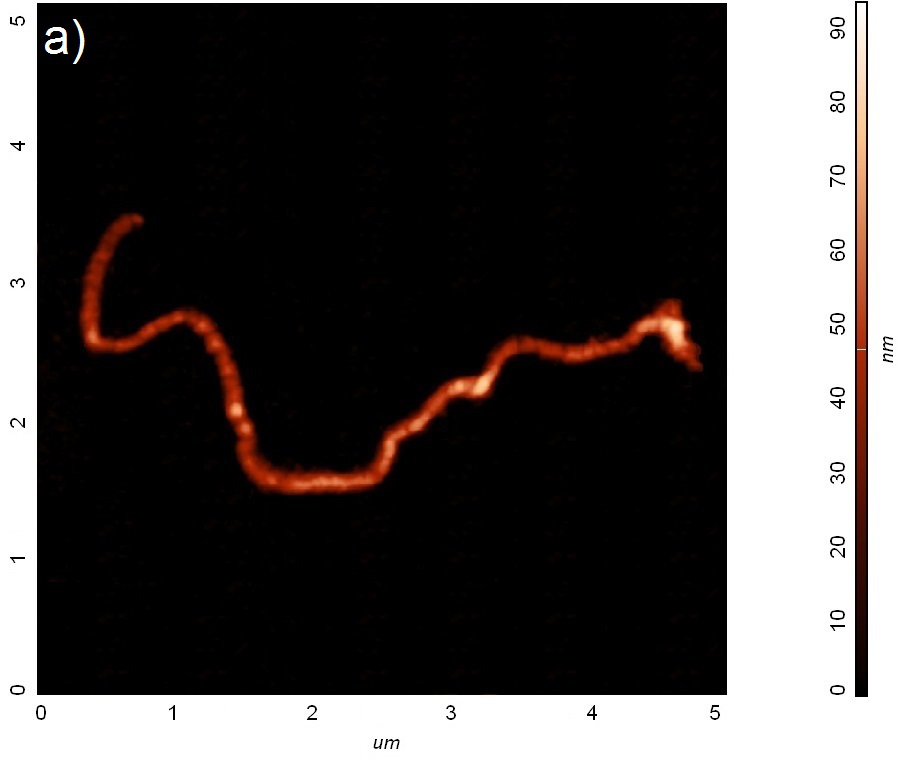 | 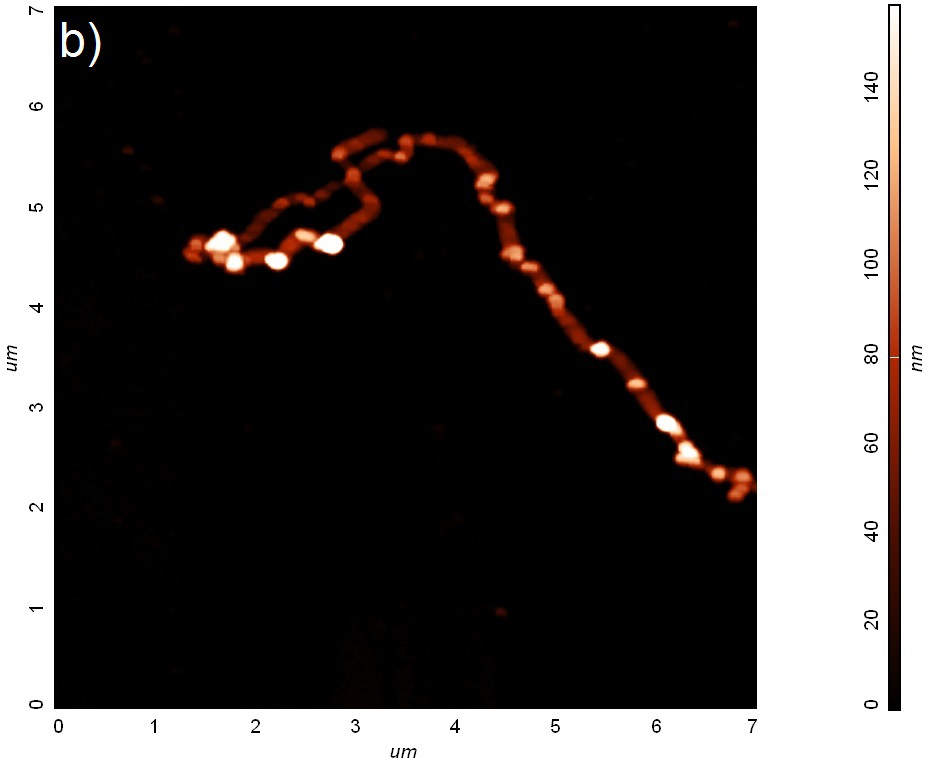 |
| --- | --- |
| 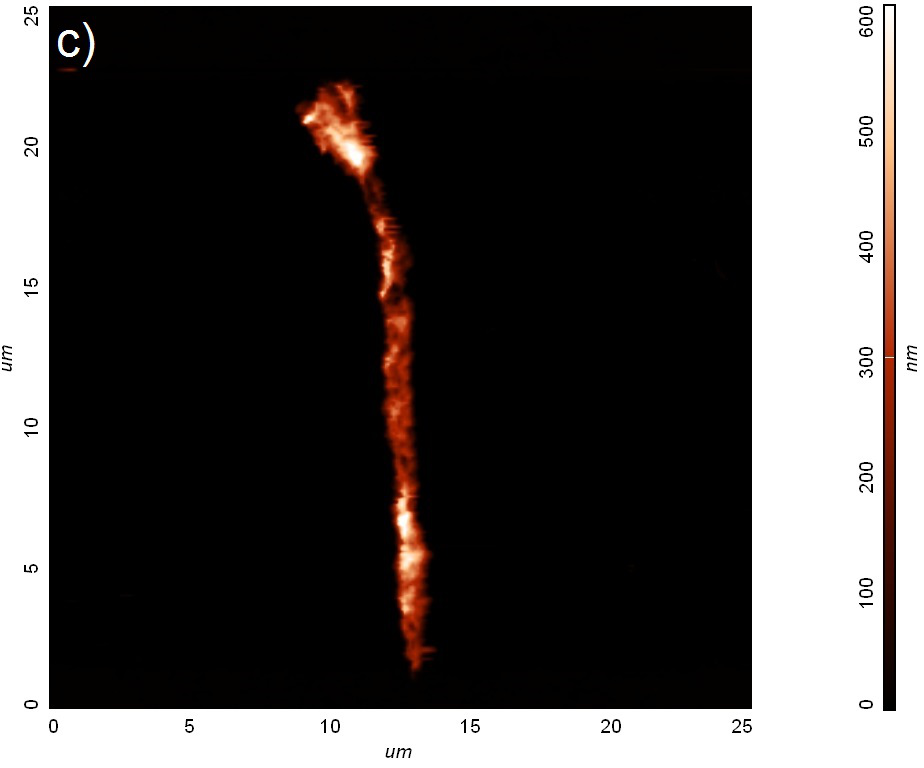 | 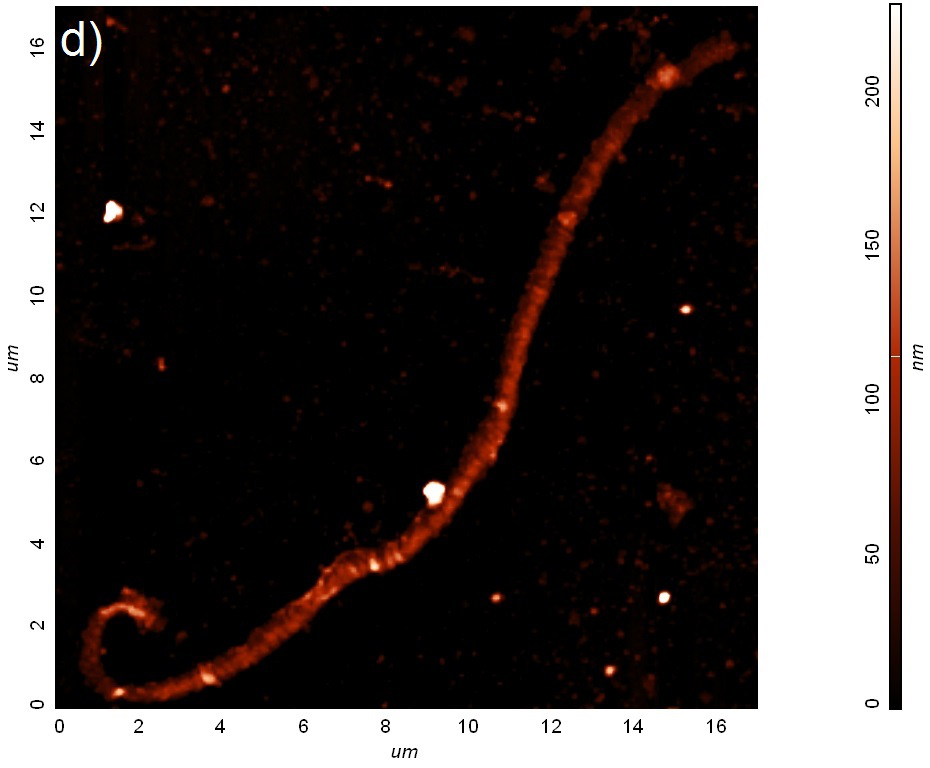 |
| 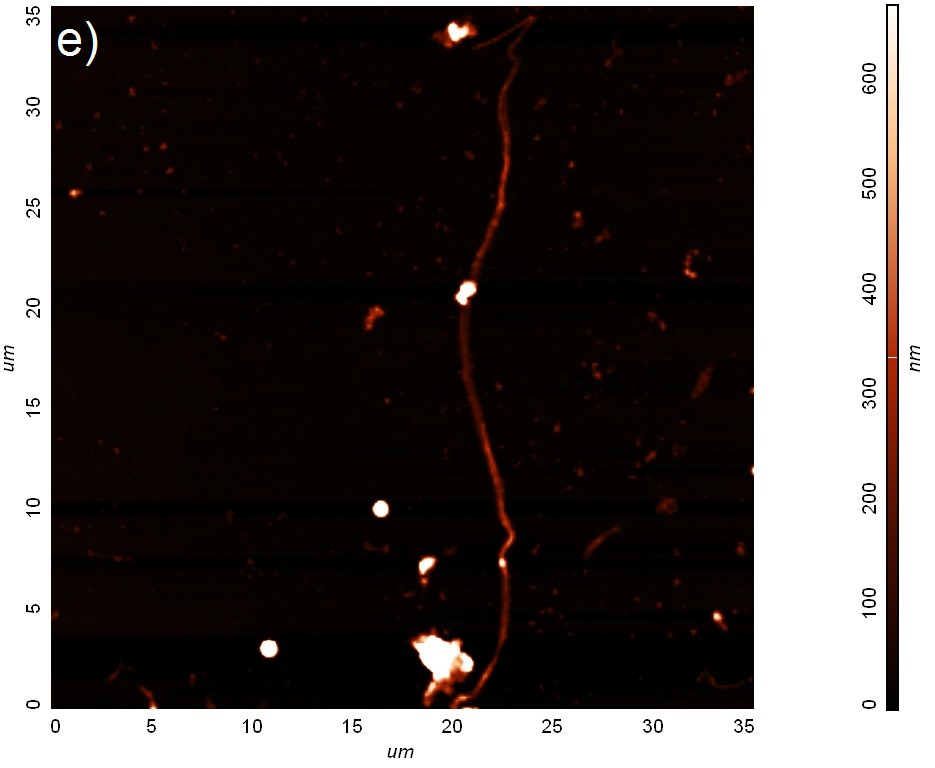 | 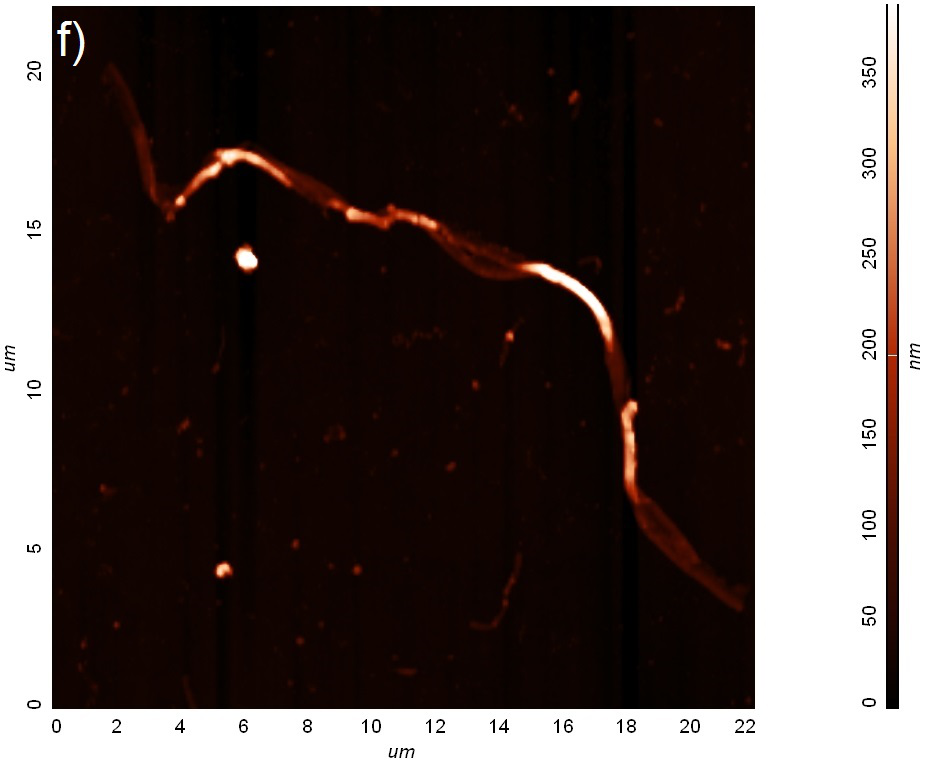 |
| **S2 Fig.** AFM topography presenting core nanofilaments of different composition: **a)** EA1, **b)** EA2, **c)** EA3, **d)** EN1, **e)** EN2, **f)** EN3. | |
